# Fabrication and Properties of a Biodegradable Zn-Ca Composite

**DOI:** 10.3390/ma16196432

**Published:** 2023-09-27

**Authors:** Mengsi Zhang, Xinyuan Wang, Shuo Zhang, Tiebao Wang, Xin Wang, Shuiqing Liu, Lichen Zhao, Chunxiang Cui

**Affiliations:** 1 Key Laboratory for New Type of Functional Materials in Hebei Province, School of Materials Science and Engineering, Hebei University of Technology, Tianjin 300130, China; 2 School of Mechanical Engineering, Hebei University of Technology, Tianjin 300130, China

**Keywords:** biodegradable metals, Zn-Ca composite, Zn-Ca intermetallic compound, cytotoxicity

## Abstract

In recent years, Zn and its alloys have become some of the most promising degradable metals as in vivo implants due to their acceptable biocompatibility and more suitable degradation rate compared with Mg-based and Fe-based alloys. However, the degradation rate of Zn-based materials after implantation in the body for orthopedic applications is relatively slow, leading to long-term retention of the implants after fulfilling their missions. Moreover, the excessive release of Zn^2+^ during the degradation process of Zn-based implants usually leads to high cytotoxicity and delayed osseointegration. To provide a feasible solution to the problem faced by Zn-based implants, a Zn-Ca composite was fabricated by an air pressure infiltration method in this work. The XRD pattern of the composite suggests that the composite is fully composed of Zn-Ca intermetallic compounds. The degradation tests in vitro show that the composite has a much higher degradation rate than pure Zn, and the high Ca content regions in the composite can preferentially degrade as sacrificial anodes. In addition, the composite can efficiently induce Ca-P deposition during immersion tests in Hank’s solution. Cytotoxicity tests indicate that L-929 cells exhibit around 82% cell viability (Grade 1) even after being cultured in the 100% extract prepared from the Zn-Ca composite for 1 day and show excellent cell viability.

## 1. Introduction

As biodegradable orthopedic implant materials for temporary implantation in vivo, Zn and its alloys have received widespread attention in recent years due to their more suitable degradation rates compared with Mg-based and Fe-based materials. However, recent studies have shown that the degradation rates of Zn-based materials after implantation in the body are relatively slow, leading to long-term retention of the implants after fulfilling their missions [1]. Meanwhile, due to the much lower tolerance values of organisms to Zn compared with that of Mg and Fe [1,2], the excessive release of Zn^2+^ of Zn-based implants during the degradation process can also lead to severe cytotoxicity in vitro and delayed bone osseointegration in vivo [1]. In view of the fact, improving the degradation rate of Zn-based implants while effectively decreasing the cytotoxicity of implants has become a research hotspot in the degradable Zn-based implant field.

Many studies found that when alloying elements such as Li [3,4,5,6,7,8,9], Sr [3,10,11], Ca [10,12,13,14,15], Y [16], Mg [16,17,18,19,20,21,22], Ti [23,24,25,26], Mn [3,12,27,28,29,30,31,32,33,34], Fe [3,35,36], Cu [3,9,23,24,25,34,37,38,39,40,41], and Ag [3,42,43,44] are introduced into Zn for alloying treatment, these alloying elements generally react with Zn to form intermetallic compounds containing Zn elements. These compounds not only significantly affect the strength and plastic deformation ability of the Zn alloys but also have a significant impact on their degradation property and cytotoxicity.

Among these alloying elements, Ca is an essential constant element for the human body. The content of Ca in the human body (1200 g) is much higher than that of other major metal elements such as K, Na, and Mg [2]. The dietary average daily intake (ADI, 743 mg) and recommended daily intake (RDI, 800–1200 mg (19–24 years old)) of Ca are also significantly higher than the corresponding values of other alloying elements such as Mg, Fe, Mn, Cu, Li, etc. [2]. Although the LD_50_ (lethal dose, 50%) of CaCl_2_ obtained via oral supplement of the mouse (2301 mg/kg) is lower than the 5000 mg/kg of MgCl_2_; the value is also significantly higher than that of other chlorides such as FeCl_3_, MnCl_2_, CuCl_2_, and LiCl [2]. Clearly, when Zn is alloyed with Ca, the dissolved Ca^2+^ during the degradation process of the alloys is difficult to cause toxicity to human cells and tissues. In addition, Li et al. [10] investigated the degradation property of the Zn-1Ca alloy in Hank’s solution, and the results showed that the Zn-1Ca alloy has a more negative corrosion potential and faster degradation rate than pure Zn in Hank’s solution. Zou et al. [13] investigated the degradation property of Zn-xCa (x = 0.5, 1, 2, 3 wt.%) alloys in Hank’s solution, and the results showed that the Zn-xCa alloys also had a more negative corrosion potential and faster degradation rate than pure Zn. Yang et al. [3] also obtained similar experimental results when they investigated the degradation behavior of Zn-xCa (x = 0.1, 0.4, 0.8 wt.%) alloys in simulated body fluids. In addition, the cytotoxicity test results conducted by Yang et al. [3] also suggested that the concentration of Zn^2+^ in the 100% extraction solution of Zn-xCa alloys decreased when the Ca content increased, and the cell activity of the alloys on MC3T3-E1 also increased accordingly.

Although the above experimental results indicate that Zn-Ca compounds in Zn-Ca alloys are beneficial to increasing the degradation rate and cell activity of Zn alloys, there are few studies that report the degradation properties, cytotoxicity, and other relevant properties of Zn-Ca compounds themselves. In view of this fact, this paper used an air compression infiltration method to prepare a Zn-Ca composite composed of Zn-Ca compounds. The microstructures and phase structures of the composite were characterized, and the mechanical properties, biodegradability, and cytotoxicity of the composite were also investigated.

## 2. Materials and Methods

### 2.1. Fabrication of a Zn-Ca Composite

**Figure 1:**
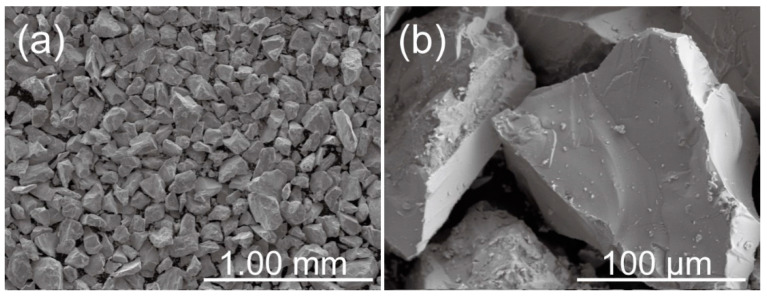
(**a**) SEM image of the Zn-Ca compound powder, (**b**) is the higher magnification of the powder.

**Figure 2:**
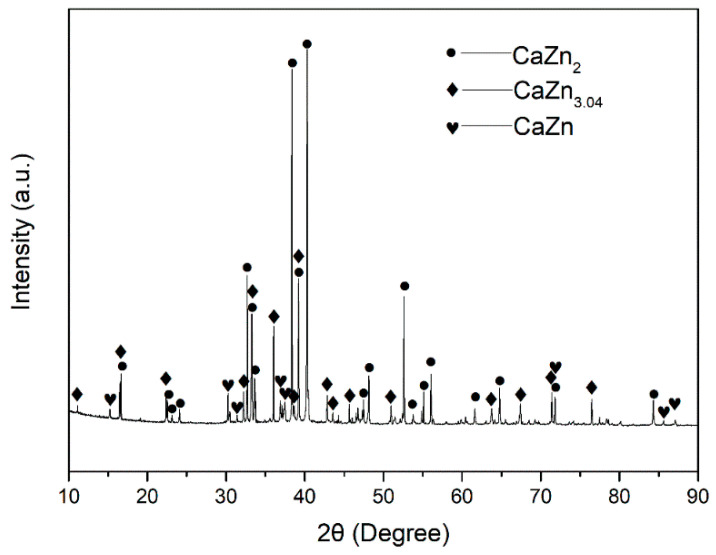
XRD pattern of the obtained Zn-Ca compound powder.

Commercially pure Zn ingot (≥99.995%) and Ca particles (≥99.0%) were used as raw materials. Zn-Ca intermetallic compounds having a nominal Zn/Ca atomic ratio of 2 were fabricated by a vacuum induction melting furnace. The obtained compounds were crushed and sieved with 100 and 200 meshes of standard sieves, and Zn-Ca compound powder with a size of around 75–150 μm was then obtained. The SEM images of the obtained Zn-Ca compound powder are shown in Figure 1. The particle size of the compounds is relatively uniform, and these particles have irregular shapes and sharp edges. The XRD pattern of the powder is presented in Figure 2. In addition to the CaZn_2_ phase, CaZn_3.04_ and CaZn phases were also found in the powder. The presence of CaZn_3.04_ and CaZn phases is due to the high viscosity of the Zn-Ca alloy melt during the preparation of CaZn_2_, resulting in uneven distribution of Ca in the alloy melt. According to the Zn-Ca phase diagram [45,46], the CaZn and CaZn_3_ phases are the most likely phases to be formed besides the CaZn_2_ phase under the current condition.

**Figure 3:**
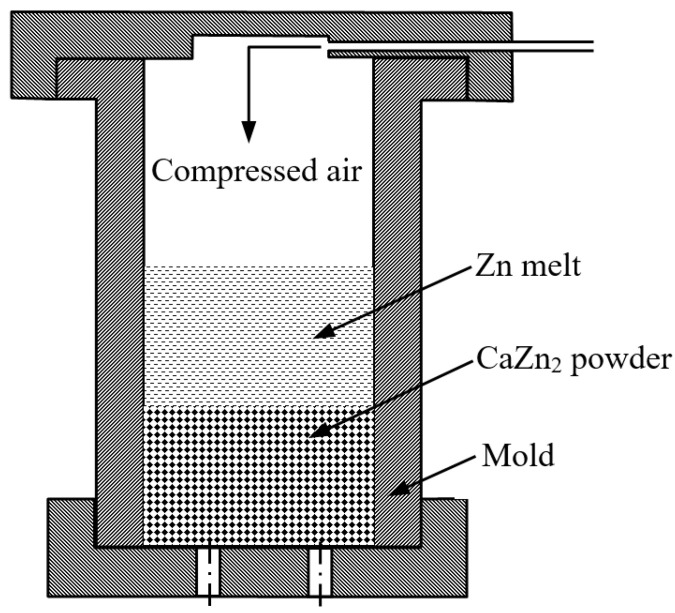
The schematic illustration of the setup for the air pressure infiltration method.

A Zn-Ca composite was fabricated by an air pressure infiltration method (APIM). The schematic illustration of the APIM setup is depicted in Figure 3. The obtained Zn-Ca compound powder was firstly filled into a steel mold with an inner diameter of 20 mm; the mold was then heated to 405 °C and held at that temperature for at least 60 min. Secondly, the Zn melt with a temperature of 560 °C was cast into the mold (the weight ratio of Zn to Zn-Ca compound powder is around 2.5:1), and the mold was quickly filled with 0.3–0.4 MPa compressed air and held for several minutes. When the mold was cooled to room temperature, the specimen was removed from the mold, and a Zn-Ca composite was obtained.

### 2.2. Microstructures and Phase Structures

The microstructures of specimens were observed by an optical microscope (Axio Imager M2m, Cari Zeiss, Oberkochen, Germany) and a scanning electron microscope (SEM, S-4800, Hitachi, Tokyo, Japan). The phase structures of specimens were characterized by an X-ray diffractometer (XRD, D8 FOCUS, Bruker, Billerica, Germany) with Cu K_α_ radiation (12°/min).

### 2.3. Compress Tests

Compressive mechanical properties of the Zn-Ca composite were characterized on a WDW-300 electronic universal testing machine (Zhongzheng, Jinan, China) at room temperature. The specimens for the tests were cut into cylinders with a size of φ4 mm × 8 mm using wire electrical discharge machining. The tests were carried out under displacement control with a crosshead speed of 1 mm/min. At least 5 specimens were tested.

### 2.4. In Vitro Biodegradation Tests

The potentiodynamic polarization curves of specimens were measured on a CHI660E electrochemical workstation using a standard three-electrode system. The testing specimen, a saturated calomel electrode (SCE), and a graphite rod served as the working electrode, the reference electrode, and the counter electrode, respectively. A Hank’s solution was employed as the corrosive medium, whose chemical composition was presented in Table 1. A 1 M HCl solution and 7.4% NaHCO_3_ solution were used to adjust the pH value of Hank’s solution to 7.4 at 37 °C. The testing specimen was first immersed in Hank’s solution at 37 °C to measure open circuit potential (OCP). When the OCP was stable, the polarization curve of the specimen was then measured at a scanning rate of 0.5 mV/s.

Immersion tests were also conducted in Hank’s solution at 37 °C. The specimens with a size of φ20 mm × 3 mm were immersed in 125 mL of Hank’s solution for different periods. After immersion, the specimens were removed from the solutions and gently washed with deionized water and then dried at room temperature. At last, the corrosion products deposited on the specimens were washed with 200 g/L CrO_3_ solution.

### 2.5. Cytotoxicity Tests

An indirect contact method was used to evaluate the cytotoxicity of the Zn-Ca composite. The employed cells were L-929 murine fibroblast cells (Cell Bank of Chinese Academy of Sciences, Shanghai City, China). The detailed process can be found in our previous work [47]. Firstly, the sterilized specimens were soaked in Roswell Park Memorial Institute (RPMI) 1640 medium containing 10% calf serum (0.2 mg/L, Zhejiang Tianhang Biotechnology Co., Ltd., Huzhou City, China) for 24 h at 37 °C. After that, the solution in the extract container was reserved as 100% extract. Secondly, 100 μL of solution having L-929 cells, whose concentration was 2 × 10^4^ cells /mL RPMI 1640 medium containing 10% calf serum, were incubated in a 96-well cell culture plate for 24 h at 37 °C in a humidified atmosphere containing 5% CO_2_. After that, 100 μL of 100%, 50%, 25%, 12.5%, and 6.25% extracts were added to the different wells of the culture plate, respectively. The negative control group was added to RPMI 1640 medium containing 10% calf serum, and the positive group was added to 10% dimethyl sulphoxide (DMSO). The culture plate was then incubated in a humidified atmosphere containing 5% CO_2_ for 24 h at 37 °C. Then, 10 μL of MTT (thiazolyl blue tetrazolium bromide) (5 mg/mL) were added to different wells and continuously incubated for 4 h. After incubation, 180 μL of DMSO were added to the wells of the plate. The optical density (*OD*) was then measured at 570 nm by a microplate reader, and the cell relative growth rate (*RGR*) was calculated by the following formula.

(1) $$RGR(\%)=\frac{OD_{\mathrm{Specimen}}}{OD_{\mathrm{Control}}}.$$

## 3. Results and Discussion

### 3.1. Microstructures and Phase Structures of the Zn-Ca Composite

**Figure 4:**
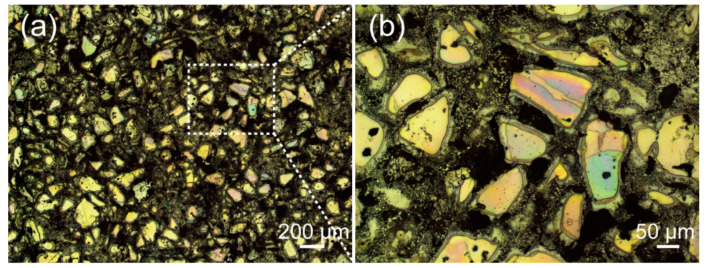
Optical images of the Zn-Ca composite, (**b**) is the higher magnification of the rectangular area in (**a**).

**Figure 5:**
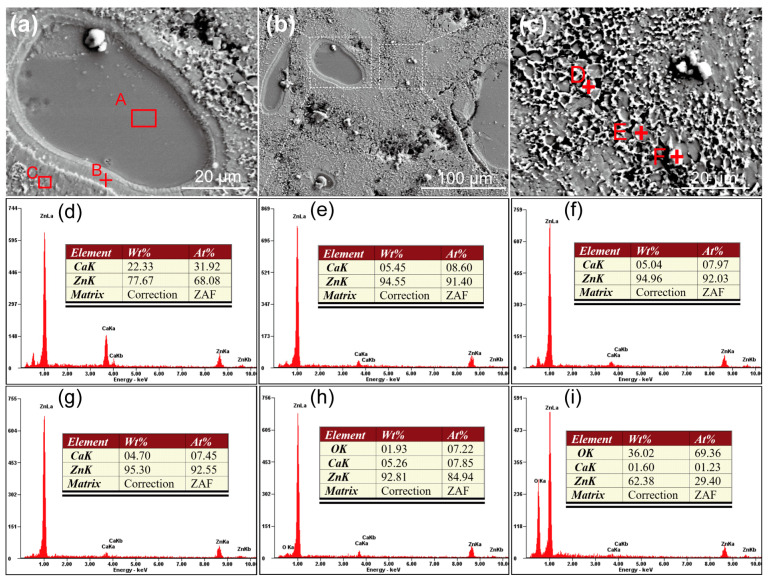
SEM images and EDS results of the Zn-Ca composite, (**a**,**c**) are the higher magnifications of the corresponding rectangular areas in (**b**), (**d**–**i**) are the EDS results of the area A, point B, and area C in (**a**) and points D, E, and F in (**c**), respectively.

The optical images of the Zn-Ca composite are shown in Figure 4. It can be seen that the particles with irregular shapes are uniformly distributed in the composite, and a shell layer is wrapped around the outer surface of many particles. The SEM images of the composite are presented in Figure 5. There is indeed a shell layer around the particles. The EDS spectrum result suggests that the atomic ratio of Ca/Zn of the particle (area A in Figure 5a) is 1:2.13, which is very close to 1:2. Therefore, it can be deduced that the particle is likely CaZn_2_. Comparing the EDS spectrum results of area A, point B, area C, and point D (Figure 5d–g), it can be found that the farther away from the particle, the lower the Ca content in the specimen. It is noted that the atomic ratio of Ca/Zn of point D (Figure 5g) is 1:12.42, which is close to 1:13. Thus, it can be deduced that the phase existing at point D may be CaZn_13_. In addition, the EDS results of points E and F suggest that the composite contains some O element, especially the white particle (point F), which has a higher oxygen content. Although the oxygen content of the specimen cannot be precisely determined by EDS, the results also indicate that the composite inevitably underwent a certain degree of oxidation during the preparation process, and further experiments are needed to investigate the effect of oxides on the properties of the composite. In addition to that, it is found in Figure 5 that the particles in the specimen are sunken compared with the surrounding region. Not only that, the shell layers around the particles also exhibit a relatively light degree of depression. Since the specimen shown in Figure 5 has been corroded by a 4% nitric acid alcohol solution, it can be determined that the particles with the highest Ca content in the specimen are the least corrosion-resistant. As the Ca content decreases, the corrosion resistance of the specimen also increases accordingly. In addition, it is also noted that the composite is not dense, and there are some pores in the specimen.

**Figure 6:**
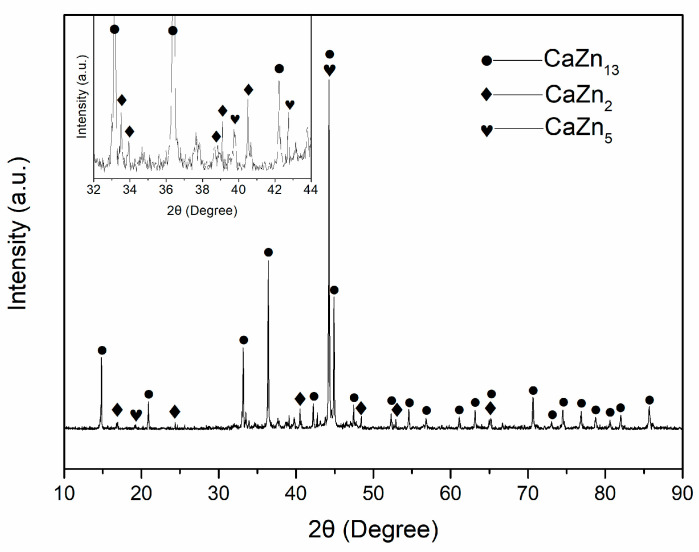
XRD pattern of the Zn-Ca composite.

The XRD pattern of the obtained Zn-Ca composite is shown in Figure 6, and the inset is a magnification pattern within the range of 32–44 degrees. The detected phases in the composite include CaZn_13_, CaZn_2_, and CaZn_5_. No Zn phase is found in the specimen, and the CaZn_3.04_ and CaZn phases existing in the original Zn-Ca compound powder are also not found. Combined with the SEM images and EDS spectra of the Zn-Ca composite in Figure 5, it can be inferred that the XRD result is due to the diffusion of Ca element from the Zn-Ca compound powder to the Zn melt as well as the formation of new phases and the disappearance of old phases during the preparation of the composite. When the Zn melt was pressed into the pores of the Zn-Ca compound powder during the infiltration process, the Ca element in the surface layer of the compound particles inevitably diffused into the surrounding Zn melt under the high-temperature action of the Zn melt. On the basis of Fick’s law, the farther away from the particles, the lower the diffused Ca content. The diffused Ca element then reacted with Zn to form new Zn-Ca compounds such as CaZn_5_ and CaZn_13_ (Figure 5g). The formation of these solid compounds not only consumed a large amount of Zn melt but also increased the resistance of Ca element within CaZn_2_ particles to diffuse outwards so that the diffusion of Ca element in the core region of the particles had not yet occurred. As a result, the core region of the particles could still retain its original high Ca content (such as CaZn_2_ shown in Figure 5a,d). On the contrary, the Ca content in the surface layer of these particles was significantly reduced (Figure 5e), and a shell layer with a low Ca content was formed around the particles (Figure 4 and Figure 5a). The disappearance of CaZn_3.04_ and CaZn phases existing in the original Zn-Ca compound powder was obviously due to the diffusion of Ca elements and the occurrence of new reactions during the infiltration process. With the diffusion of the Ca element and the reactions of Ca with Zn, the Zn in the pores of the Zn-Ca compound particles was almost depleted, which ultimately led to the absence of the Zn phase in the XRD pattern of the composite. As a result, the obtained composite is entirely composed of Zn-Ca compounds.

### 3.2. Compressive Mechanical Properties of the Zn-Ca Composite

**Figure 7:**
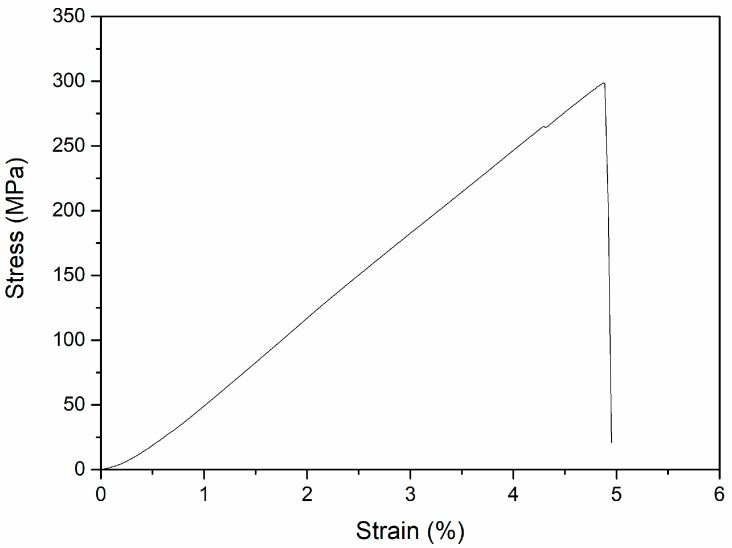
Typical compressive stress–strain curve of the Zn-Ca composite.

The compressive stress–strain curve of the Zn-Ca composite is depicted in Figure 7. Unlike as-cast pure Zn [10,48] and some Zn alloys such as Zn-1Ti [49], Zn-1X (X = Mg, Ca, Sr) [10], Zn-2Mg [50], and Zn-3Cu [50] that have good compressive plasticity, the composite exhibits typical brittle fracture characteristics. Clearly, the result can be attributed to the fact that both the matrix and reinforcing phases of the composite are Zn-Ca compounds. The calculated mean compressive strength of the composite is 281.1 ± 55.3 MPa. The compressive strength value is obviously not high among the Zn alloys that also have brittle fracture characteristics (e.g., Zn-3Mg-1Ti alloy with eutectic structure has a compressive strength of 625.1 MPa [49]), which is due to the fact that the specimen is not dense (Figure 5). Since no extensometer was used during the compressive process, the elastic modulus of the specimen was not calculated.

### 3.3. In Vitro Biodegradable Properties of the Zn-Ca Composite

**Figure 8:**
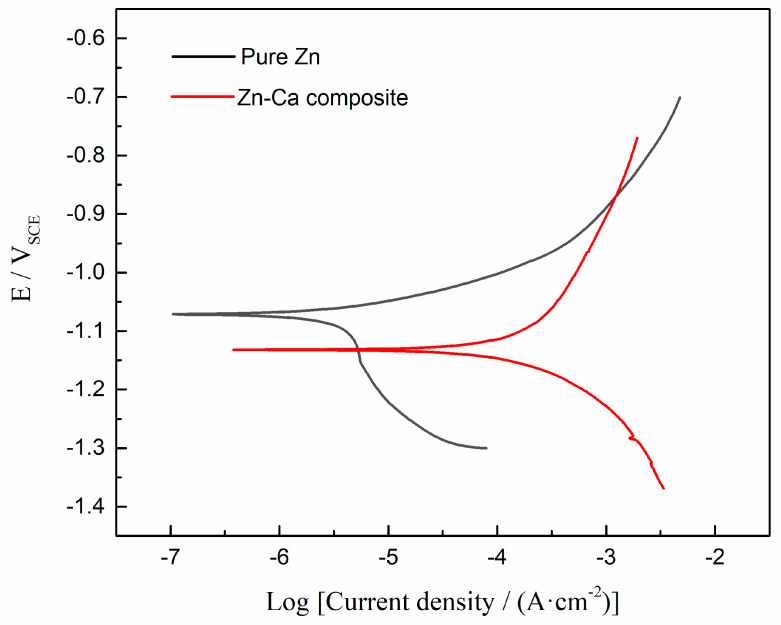
Potentiodynamic polarization curves of the Zn-Ca composite and pure Zn.

The potentiodynamic polarization curve of the Zn-Ca composite is shown in Figure 8. For comparison, the polarization curve of an as-cast pure zinc is also plotted in Figure 8. The corrosion potentials (*E*_corr_) and the corrosion current densities (*I*_corr_) derived from the polarization curves are listed in Table 2. Clearly, the corrosion potential of the Zn-Ca composite is more negative than that of pure Zn. Moreover, the composite also exhibits a higher degradation rate than pure Zn.

**Figure 9:**
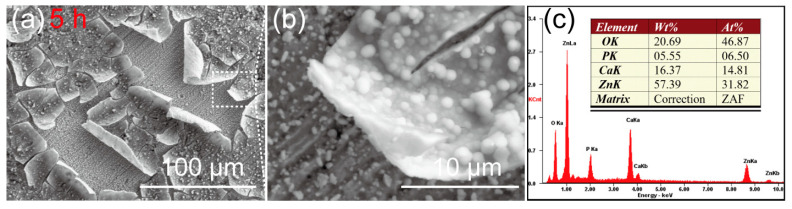
(**a**,**b**) SEM images of the Zn-Ca composite after immersion in Hank’s solution for 5 h, (**b**) is the higher magnification of the rectangular area in (**a**), (**c**) is the EDS result of the area shown in (**b**).

**Figure 10:**
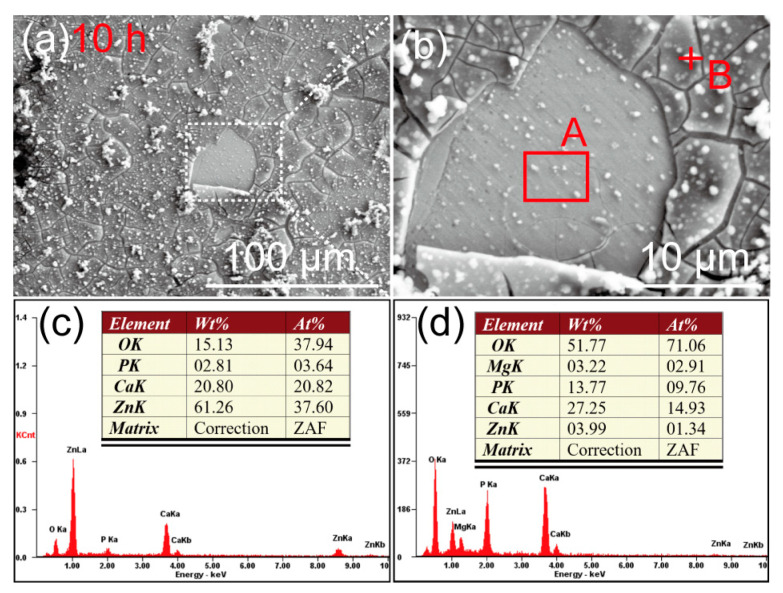
(**a**,**b**) SEM images of the Zn-Ca composite after immersion in Hank’s solution for 10 h, (**b**) is high magnification of the white rectangular area in (**a**); (**c**,**d**) are the EDS results of area A and point B in (**b**), respectively.

The SEM images of the Zn-Ca composite after immersion in Hank’s solution for 5 h are shown in Figure 9. A thin layer of corrosion products composed of spherical particles has been deposited on the Zn-Ca composite. Due to the dehydration of the specimen during the drying process after immersion, the corrosion product layer was warped. Nevertheless, it still can be inferred that the surface of the specimen has been completely covered by the corrosion product layer after immersion for only 5 h. Under the warped corrosion product layer, the original scratches on the specimen surface after sanding and polishing can still be seen, although some corrosion product particles were deposited. The EDS spectrum result (Figure 9c) suggests that the corrosion product layer is rich in Zn, O, Ca, and P elements. Figure 10 shows the SEM images of the Zn-Ca composite after immersion in Hank’s solution for 10 h. Although the corrosion product layer also cracked, the cracked corrosion product layer did not undergo severe warping. This result is mainly due to the thickening of the corrosion product layer. In addition, the corrosion product layer on the specimen surface has partially detached, and there is still corrosion product deposition on the exposed surface. Unlike after immersion for 5 h (Figure 9), the original scratches on the exposed surface of the partially detached corrosion product layer are no longer obvious. The EDS result of the exposed surface (area A in Figure 10b) suggests that the surface is still rich in Zn, O, Ca, and P elements. However, the EDS spectrum of point B in Figure 10b shows that the corrosion product layer is rich in O, Ca, and P as well as a small amount of Mg, while the Zn content is very low. This result further indicates that the corrosion product layer deposited on the composite surface is already thick enough to prevent the collection of Zn information from the specimen itself after soaking for 10 h. 

**Figure 11:**
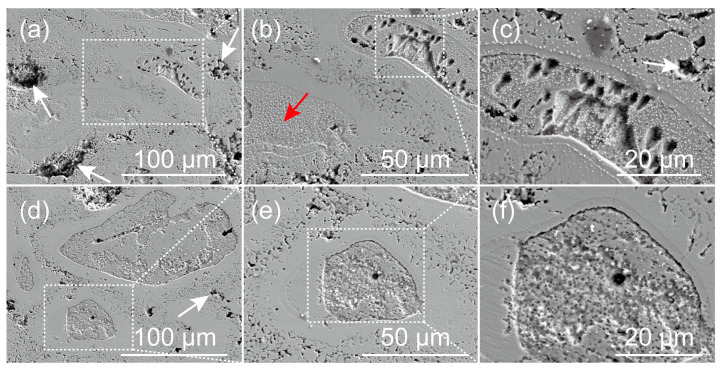
SEM images of the Zn-Ca composite after immersion in Hank’s solution for 5 h (**a**–**c**) and 10 h (**d**–**f**) and then removal of corrosion products with chromic acid solution.

The SEM images of the Zn-Ca composite after immersion in Hank’s solution for 5 h and 10 h and then removal of corrosion products with chromic acid solution are shown in Figure 11. After immersion for only 5 h, obvious localized corrosion was observed on the surface of the specimen. Corrosion pits were mainly found in the core regions of the reinforcing particles with core–shell structures. As seen in Figure 11a–c, the core regions of some particles with core–shell structures were heavily corroded. However, there are also particles where the core regions are not severely corroded (red arrow in Figure 11b). In addition, it should be noted that the pits present between the reinforcing particles (white arrows in Figure 11) were not caused by localized corrosion but were formed during the preparation process of the specimen. After immersion for 10 h, the core regions of many reinforcing particles have corroded off a layer and formed obvious depressions. Unlike the core regions, the shell layers surrounding these regions and their outer regions (Zn-rich region) still did not undergo significant degradation. However, it can be inferred that these Zn-rich regions will also undergo serious corrosion with the extension of immersion time.

The reason for the above phenomena can be attributed to the preferential degradation of the local regions with more negative corrosion potential in the specimen as sacrificial anodes. It is known that the standard electrode potential of Ca is −2.87 V_SHE_, which is significantly lower than that of Zn (−0.762 V_SHE_). Therefore, it can be inferred that the electrode potentials of Zn-Ca compounds are also lower than that of Zn. Now, the inference has been confirmed by the polarization curves shown in Figure 8. Similarly, MgZn_2_ and Mg_2_Zn_11_ produced by the reaction of Zn with the element Mg, which also has a more negative standard electrode potential than Zn (−2.372 V_SHE_ vs. −0.762 V_SHE_), have a more negative corrosion potential than Zn in the corrosion solution [51]. Then, it can be further inferred that the Zn-Ca compound having a higher Ca content, such as CaZn_2_, will have a more negative corrosion potential. As the Ca content in the compound decreases, the corrosion potential of the compound will be closer to that of Zn. Consequently, corrosion couples will be formed between the CaZn_2_ core regions and their shell layers, as well as the shell layers and their outer matrix (Ca content is relatively lower) due to the difference in corrosion potential, ultimately leading to the preferential degradation of the core regions that have the most negative corrosion potential. As for the shell layers around the core regions did not show obvious degradation compared with their outer matrix during soaking for 5 h and 10 h; this result is mainly due to the small difference in Ca content between the shell layers and its outer matrix (Figure 5). Clearly, the potential difference between the two electrodes constituting a corrosion couple significantly affects the degradation of the sacrificial anode. When the potential difference between the two electrodes is small, it will take a longer time to observe significant degradation of the sacrificial anode.

The preferential degradation of the sacrificial anodes (the core regions of the reinforcing particles) not only led to severe localized corrosion but also made the degradation rate of the Zn-Ca composite significantly higher than that of pure Zn (Figure 8 and Table 2). The corrosion current density of the Zn-Ca composite is almost 57.5 times that of pure Zn. However, the corrosion current densities of the Zn-0.8Ca, Zn-1Ca, and Zn-2Ca alloys measured by Yang et al. [3], Li et al. [10], and Zou et al. [13] are only 1.23, 1.19, and 2.33 times that of pure Zn, respectively. The result is clearly attributed to the fact that the Zn-Ca composite is entirely composed of Zn-Ca compounds. 

In addition, the preferential degradation of the sacrificial anodes also accelerated the cathodic reaction (2) occurring on the protected cathodes, thereby significantly increasing the pH value of the soaking solution.

2H_2_O + O_2_ + 4e → 4OH^−^. (2)

Koji et al. [52] measured the pH change in Hank’s solution soaking of bulk CaZn_2_ during immersion tests. After 24 h of immersion, the pH value of the solution was basically stabilized at approximately 8.7. Zhao et al. [53] believed that the OH^−^ ions produced by the reaction (2) are essential for inducing the deposition of Ca and P elements on biodegradable Zn-based materials. Moreover, the higher the pH of the soaking solution, the more favorable the deposition of calcium phosphates [54]. Although the pH value of Hank’s solution is only 8.7, measured by Koji et al. [52], the pH value of Hank’s solution soaking of the Zn-Ca composite should possess a higher pH value due to the accelerated reaction (2) on the protected cathodes. The higher the pH value of the soaking solution, the greater the amount of OH^−^ ions accumulated on the surface of the composite should be. Thus, the compounds containing Ca and P elements are more easily deposited on the surface of the Zn-Ca composite. As a result, a corrosion product layer containing Ca and P elements was deposited on the Zn-Ca composite only after 5 h of immersion in Hank’s solution (Figure 9). For the porous Zn scaffold, only some tiny corrosion products were dispersedly deposited on the porous Zn scaffold even after 1 d of immersion in Hank’s solution under the same condition [55]. It can be seen that the Zn-Ca composite is far easier to induce Ca and P deposition and exhibits excellent bioactivity. 

### 3.4. Cytotoxicity of the Zn-Ca Composite

**Figure 12:**
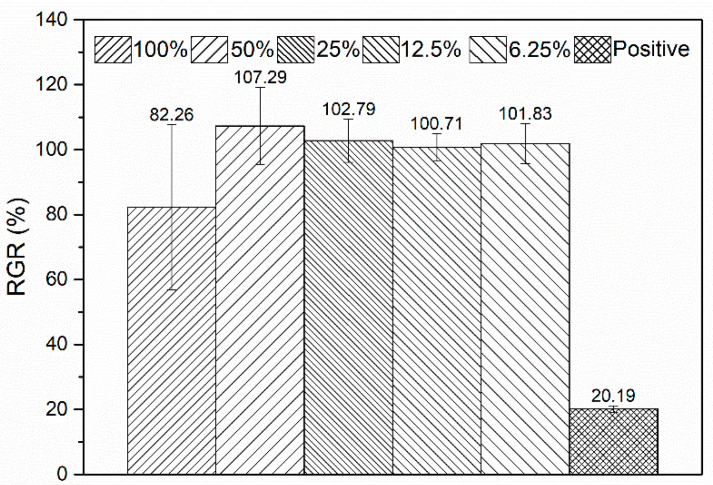
L-929 cell viability after incubation in extracts with different concentrations for 1 day.

The cell viability of L-929 after incubation in the 100%, 50%, 25%, 12.5%, and 6.25% extracts for 1 d is presented in Figure 12. It can be seen that the cell viability of L-929 after culturing in the 100% extract reaches 82.26 (Grade 1) and shows excellent viability. When the 100% extract is diluted to 50%, 25%, 12.5%, and 6.25%, the cell viability of L-929 is higher than 100% (Grade 0). Clearly, these extracts show no cytotoxicity to L-929 cells. Generally, the cytotoxicity of the extracts prepared from biodegradable Zn-based materials is closely related to the concentration of Zn^2+^ in them. Although different cells have different tolerance concentration thresholds to Zn^2+^, the thresholds are generally very low. For example, Yang et al. [56] reported that the ZnCl_2_ solution containing 23.9 μg/mL of Zn^2+^ exhibited severe cytotoxicity on MC3T3-E1 cells, while 12.1 μg/mL of Zn^2+^ significantly promoted cell proliferation. He et al. [57] also reported that when the Zn^2+^ concentration in ZnCl_2_ solution was lower than 19.62 μg/mL, the solution showed good cell viability on MC3T3-E1 cells. However, when the Zn^2+^ concentration increased to 29.43 μg/mL, the cell viability decreased to around 3.7%. For the L-929 cells, the highest safe concentration of Zn^2+^ is only 5.233 μg/mL, as reported by Kubásek et al. [22]. Although Zn metal has a relatively slow degradation rate, the released Zn^2+^ ions during degradation are still excessive to cells. As a result, pure Zn [56,58,59] and other Zn-based implants such as Zn-4Cu alloy [40] and porous Zn scaffold [55] usually exhibit pronounced cytotoxicity in vitro. When these materials with in vitro cytotoxicity are implanted into the body, a layer of fibrous connective tissue is often observed around the implants [56,58,60,61,62]. The connective tissue layer prevents new bone from directly bonding to the implant, resulting in delayed osteointegration.

Although the Zn^2+^ concentration in the 100% extract prepared from the Zn-Ca composite was not measured, it still can be inferred that the released Zn^2+^ concentration should not be high during the degradation. Clearly, this result is attributed to the protective effect of the sacrificial anodes with high Ca content on the cathodes with high Zn content. In addition, Yang et al. [3] pointed out that the appropriate content of Ca is helpful in eliminating the toxicity of Zn. Therefore, the dissolved Ca^2+^ during the degradation of the Zn-Ca composite may also play a certain role in improving the cell viability of the material. Further experiments are required to verify the inference. 

## 4. Conclusions

A Zn-Ca composite was successfully fabricated by an air pressure infiltration method using Zn-Ca intermetallic compound powder and Zn ingot. The microstructure, phase structures, compressive mechanical properties, in vitro degradation properties, and cytotoxicity of the composite were investigated. The main conclusions are as follows:

(1) The Zn-Ca composite is fully composed of Zn-Ca intermetallic compounds. The reinforcing phases are Ca-rich particles with core–shell structures, while the matrix phases are rich in Zn elements.
(2) The composite exhibits typically brittle fracture characteristics during compressive tests, and the compressive strength of the composite is around 281.1 MPa.
(3) The core regions of the reinforcing particles degrade preferentially as sacrificial anodes, resulting in severe localized corrosion of the composite during immersion tests. The preferential degradation of the sacrificial anodes also makes the composite have a much faster degradation rate than pure zinc and a better ability to induce Ca and P deposition than pure Zn in Hank’s solution.
(4) The preferential degradation of the Ca-rich core regions also provides the composite with excellent cell viability.

In conclusion, the utilization of Ca-rich Zn-Ca compounds as sacrificial anodes accelerates the degradation of the Zn-based implant while also maintaining excellent cell viability. This strategy provides a new pathway for the preparation of degradable Zn-based implants with relatively fast degradation rates as well as good biocompatibility. 

## Figures and Tables

**Table 1:** Chemical composition of Hank’s solution.

Number	Reagent	Concentration
1	CaCl_2_	0.14 g/L
2	NaCl	8.00 g/L
3	KCl	0.40 g/L
4	NaHCO_3_	0.35 g/L
5	Glucose (C_6_H_12_O_6_)	1.00 g/L
6	MgSO_4_∙7H_2_O	0.06 g/L
7	KH_2_PO_4_	0.06 g/L
8	Na_2_HPO_4_∙12H_2_O	0.06 g/L
9	MgCl_2_∙6H_2_O	0.10 g/L

**Table 2:** Corrosion potentials and corrosion current densities derived from the polarization curves.

Specimens	*E*_corr_ (V_SCE_)	*I*_corr_ (μA/cm^2^)
Pure Zn	−1.070	3.98
Zn-Ca composite	−1.131	229

## Data Availability

The data presented in the manuscript will be available on request from the corresponding author.
